# Prevalence and risk factors of gestational diabetes mellitus in a population of pregnant women attending three health facilities in Limbe, Cameroon: a cross-sectional study

**DOI:** 10.11604/pamj.2018.31.195.17177

**Published:** 2018-11-20

**Authors:** Thomas Obinchemti Egbe, Elvis Songa Tsaku, Robert Tchounzou, Marcelin Ngowe Ngowe

**Affiliations:** 1 Department of Obstetrics and Gynecology, Faculty of Health Sciences, University of Buea, Cameroon; 2 CMA Sonjkolong, Adamawa Region, Cameroon; 3 Department of Surgery, Faculty of Health Sciences, University of Buea, Cameroon

**Keywords:** Gestational diabetes mellitus, IADPSG, macrosomia, oral glucose tolerance test

## Abstract

**Introduction:**

There are few studies regarding gestational diabetes mellitus (GDM) in the South West Region of Cameroon. We aimed at determining the prevalence and risk factors of GDM in three health facilities in the Limbe health district, Cameroon.

**Methods:**

A cross-sectional study was carried out in one secondary, and two primary healthcare facilities in Limbe, Cameroon during the period 1^st^ November 2016 to 31^st^ January 2017. We administered a pretested questionnaire on 200 consenting pregnant women at 24-28 weeks' gestation. We carried out a 2-hr oral glucose tolerance test after fasting overnight. GDM was diagnosed when ≥1 plasma glucose (PG) test result was abnormal according to the IADPSG criteria (FPG ≥92 mg/dL, PG 1-hr 180mg/L, PG 2-hr 153 mg/dL). Data analysis was with Epi-Info^TM^ version 3.5.4. Associations were analyzed with the Pearson's chi squared and Fischer's exact test where appropriate. Statistical significance was set at p < 0.05.

**Results:**

The prevalence of GDM was 20.5% and respondents' mean age was 27.8 (SD 5.7) years. Majority, 13.5% participants had abnormal FPG alone, while 3.5% had any two abnormal values. GDM was associated with: advanced maternal age (OR 3.4: 95% CI 1.7-7.0; P<0.001), BMI≥30 kg/m^2^ (OR 6.2 : 95% CI 2.9-13.1, P<0.001), past history of unexplained stillbirth (OR 5.7: 95% CI 2.5-12.9, P<0.001) and history of macrosomia (OR 8.5:95% CI 3.8-19, P<0.001).

**Conclusion:**

With the high prevalence of GDM, identification of its associated factors has the potential to be a target of intervention to prevent poor obstetrical outcomes.

## Introduction

The prevalence of diabetes, especially gestational diabetes mellitus (GDM), is increasing globally. Gestational diabetes mellitus is any degree of glucose intolerance leading to a hyperglycemic state of variable severity, first recognition during pregnancy, no matter the treatment required or postpartum evolution [1, 2]. The true prevalence of GDM is unknown, but it has been estimated in the United States of America to vary from 1% to 14% of pregnancies, depending on the population studied and the diagnostic tests used [3, 4].

GDM complicates approximately 4% of all pregnancies and women with it have an approximate 7-fold risk of developing type-2 diabetes mellitus in the future, as well as their children and subsequent generations [5]. This fact should alert the obstetrician to the necessity to pay special attention to this segment of the population, especially in low-income countries [3, 6]. The impact of GDM on maternal and fetal health has been increasingly recognized [7]. GDM increases the risk of fetal macrosomia, which is associated with secondary complications like shoulder dystocia, cesarean delivery and birth trauma. There is also a concomitant increase in neonatal complications like hypoglycemia, respiratory distress syndrome, hypocalcemia and hyperbilirubinemia [8]. Risk assessment for GDM should be done at the first antenatal care visit [9]. Competing diagnostic criteria across the globe have complicated healthcare, the design and interpretation of research on GDM. The Hyperglycemia and Adverse Pregnancy Outcomes (HAPO) study was intended to lead to unification and agreement on the diagnostic criteria for GDM [7, 10]. Furthermore, in 2010, the International Association of Diabetes in Pregnancy Study Groups (IADPSG) released their recommendation for a new set of diagnostic criteria based on HAPO study outcomes: 92 mg/dL, 180 mg/dL and 153 mg/dL for the fasting plasma glucose (FPG), 1-hr and 2-hr PG levels, respectively [10]. The goal standard for testing GDM is the Oral Glucose Tolerance Test (OGTT) at 24 to 28 weeks' gestation after a 75 g oral glucose loading dose on a fasting subject as has previously been described [1]. Therefore, fasting is defined as no caloric intake for ≥8 hours [9].

It has been reported that the global trend of an increased prevalence of diabetes in African populations and the subsequent increase of diabetes in pregnancy is closely linked to the increase in obesity [11]. In a study of 11 568 pregnant women in six regions of Cameroon, Sobngwi et *al*. (2010) reported a 5% to 17% prevalence of GDM [12]. Another study in Cameroon reported a 6.3% (20/316) prevalence of GDM and that in multivariate analysis older age (≥30 years) remained a significant predictor of GDM [13]. Munang et *al*. (2017) reported that in a group of 84 pregnant Cameroonian women, the 75g OGTT was reproducible only in 74.2% cases and that maternal age and BMI were factors associated with non-reproducible results [14]. Despite the potential adverse impact GDM may have on pregnancy and its outcome, the prevalence and risk factors (advanced maternal age, body mass index (BMI), history of previous stillbirths, large for gestational age (LGA) and macrosomia, family history of diabetes mellitus, history of congenital malformations, past history of GDM and polycystic ovarian syndrome (PCOS) etc) of this condition have not yet been adequately studied in the LHD. To our knowledge, no epidemiologic study regarding GDM has been carried out in the Limbe Health District, Cameroon.

This study will help bridge the gaps regarding the prevalence and risk factors of GDM in a population of pregnant women attending three hospitals in the Limbe Health District; namely, the Limbe Regional Hospital, CMA Limbe and Bota District Hospital. We hypothesize that there is a high prevalence of GDM in the Limbe Health District in Cameroon and that this prevalence will increase significantly given the known risk factors. The aim of this study was to determine the prevalence and risk factors of gestational diabetes mellitus in three health facilities in the Limbe health district.

## Methods

### Study design and setting

During the period 1^st^ November 2016 to 31^st^ January 2017, we carried out a cross sectional study in three health facilities in the LHD of the South West Region of Cameroon; namely, the LRH, a secondary healthcare facility; CMA Limbe, and the Bota District Hospital, two primary healthcare facilities. The Limbe Health District (LHD) is made up of eight health areas which include: Bota, Mabeta, Idenau, Bojongo, Sea Port, Moliwe, Batoke and Zone II. The LHD is wedged between Mount Cameroon to the North and the Atlantic Ocean to the south, and its health facilities are equipped to take care of pregnant women.

### Study population and sampling

Eligible subjects were consenting pregnant women between 24-28 weeks of gestation who came for antenatal care visits in the three health facilities (LRH, CMA Limbe and BDH) during the study period. Participants were enrolled by convenient and consecutive sampling methods.

### Sample size determination

Using a 95% confidence interval, power of 80% and a proportion (p) of gestational diabetes from a previous study in Cameroon of 5%, Sobngwi *et al*. 2010, (p=0.05) and using the formula for sample size estimation [15], we calculated a sample size of 73 participants. However, we enrolled 200 participants for study.

### Study procedure

After obtaining ethical clearance from the Institutional Review Board of the Faculty of Health Sciences of the University of Buea (Ref: 2016/141/UB/FHS/IRB) and administrative authorization from the Regional Delegation of Public for the South West Region (Ref: R11/MINSANTE/SWR/RDPH/PS/4887/223), we enrolled 200 pregnant women who came for antenatal care services at 24-28 weeks' gestation and signed a written informed consent form. We respected the principles of the Helsinki Declaration in this study [16].

### Questionnaire and interview

A 20-item questionnaire was developed from pre-existing questionnaires [17] and approved by the University of Buea Institutional Review Board. It was beta-tested on 10% of the total sample, a similar population, one week before the commencement of the study, for comprehension and content validity. The surveyors were trained on all aspects of the study including exclusion criteria. A total of 200 questionnaires were to be administered on consenting participants. The questionnaires were both interviewer or self-administered and were designed to take 15-20 minutes to administer. The questionnaire covered the participant's socio-demographic data, obstetric history (gravidity, parity, stillbirths, abortions, number of live births, previous birth weights), history of congenital malformations, past history of diabetes, family history of diabetes. There was also a section in the questionnaire for the procedures performed: the oral glucose tolerant test according to the International Association of Diabetes and Pregnancy Study Group (IADPSG) criteria as already described [10]. Each participant was handed her results in a sealed envelop at the end of the study. Those whose results were positive were counseled and referred to see a diabetologist or a specialist in internal medicine (in the absence of a diabetologist) for follow-up in collaboration with the obstetrician.

### Data management and data analyses

The data was coded, double-checked and entered into Microsoft excel 2010. Data was then exported to a predesigned template in Epi info^TM^ 3.5.4 and analysed. In computing the socio-demographic characteristics of study participants, measures of central tendencies (mean, standard deviation and interquartile ranges) were used while frequencies were used to compute level of education, religion, marital status, occupation, etc. The predictor variables were maternal age, past history of stillbirths, major congenital malformations, and macrosomia or large babies ≥4000 g and a family history of DM. The outcome variable was the presence of GDM. Participants were classified as having GDM or not, based on the IADPSG criteria. The Chi squared and Fischer's exact tests were used to test for significant associations. Bivariate analysis was done and results were reported as p-values, odds ratios (OR) and their 95% confidence intervals (CI). Statistical significance was set at P<0.05.

## Results

### Socio-demographic characteristics

A total of 200 pregnant women were enrolled into the study and 41 (20.5%) had GDM. Majority, 13.5% (27/200) participants had abnormal FPG alone, while 2% (4/200) had all plasma glucose (PG) values abnormal and 3.5% (7/200) had any two abnormal (PG) values. The mean age of study participants was 27.8 (SD 5.7) years; range 16 to 42 years. Most, 31.5% (63/200) participants were in the age group 21-25 years while 31% (62/200) were in the age group 26-29 years. Majority, 47.5% (95/200) participants were self-employed while 30.5% (61/200) were unemployed. 33.5% (67/200) and 31% (63/200) participants were from the North West and South West Regions respectively. Foreigners represented only 3.5% (7/200) of study participants; mainly from Nigerians and Ghanaians. Furthermore, 65% (130/200) participants had secondary level of education and 21.5% (43/200) had tertiary level of education. 0.5% (1/200) participants did not have any formal education. Majority, 61% (122/200) of the participants were married (Table 1).

**Table 1:** Socio-demographic characteristics of study population

	Frequency (N=200)	Percentage
**Blood glucose parameters**		
Normal	159	79.5%
Abnormal FPG only	27	13.5%
Abnormal FPG, 1^st^ and 2^nd^ hour	04	02.0%
Abnormal Any two values	07	03.5%
Abnormal 1^st^ or 2^nd^ hour	03	01.5%
**Age**		
16-20	18	09.0%
21-25	63	31.5%
26-29	62	31.0%
>30	57	28.5%
**Occupation**		
Self employed	95	47.5%
Civil servant	20	10.0%
Student/Unemployed	85	42.5%
**Education**		
Primary Secondary	27 130	13.5% 65.0%
Tertiary	43	21.5%
**Marital status**		
Single	69	34.5%
Married	122	61.0%
Divorce/Widow	09	04.5%

**FPG**: fasting plasma glucose

### Obstetric and medical history of study participants

The mean gestational age (GA) of participants was 26.2 (SD 1.4) weeks; range 24-28 weeks. Most, 47% (94/200) respondents were between 27-28 weeks gestation while 14.5% (29/200) were at 24 weeks gestation (Table 2). The mean gravidity was 3 (SD 1.5); range 1-6. There were 68.5% (137/200) participants whose gravidity was between 2-4 and 15% (30/200) were grand multigravida with ≥5 births. There were only 4.5% (9/200) participants with preterm births. The mean number of abortions, both induced and spontaneous, was 0.6 (SD 0.7). Nonetheless, 45% (90/200) participants had one to three abortions; among which 3% (6/200) had 3 abortions and 36.5% (73/200) had one abortion. Moreover, the mean number of spontaneous abortions was 0.3 (SD 0.5) and 24% (48/200) participants had a history of one to two spontaneous abortions; 23% (46/200) had one spontaneous abortion and 1% (2/200) had two spontaneous abortions. About, 16% (32/200) participants had a history of unexplained stillbirth, 18% (36/200) also had a history of macrosomia ≥4000 g. The mean BMI was 38.8 (SD 3.8) kg/m^2^; mode 27.6 Kg/m^2^; range; 19.1 to 43.5 kg/m^2^. Nonetheless, 52% (105/200) participants were overweight (BMI 25-29.9 Kg/m^2^) while 34.5% (69/200) participants were obese (BMI>30 Kg/m^2^). There were also 1% (2/200) with a history of congenital malformations (one case of imperforate anus and another with limb defects; 37.5% (75/200) with a family history of diabetes mellitus with 16.6% (33/200) from first degree relatives; 8% (16/200) with chronic diseases, mainly HIV 6.5% (13/200) and hypertension 1.5% (3/200), both receiving treatment (Table 3).

**Table 2:** Obstetric history of study participants

Variable	Frequency (N=200)	Percentage (%)
**Gestational age (weeks)**		
24 -26 weeks	106	53%
27-28 weeks	94	47%
**Gravidity**		
1	33	16.5%
2-4	137	68.5%
≥5	30	15.0%
**History of preterm delivery**		
Yes	09	04.5%
No	191	95.5%
**History of spontaneous abortions**		
0	152	76.0%
1-2	48	24.0%
**History of unexplained stillbirth**		
Yes	32	16.0%
No	168	84.0%
**History of macrosomia**		
Yes	36	18.0%
No	164	82.0%
**Body mass index (Kg/m2)**		
18.5-24.9	26	13.0%
25.0-29.9	105	52.5%
>30	69	34.4%

**>: Greater than; ≥:** Greater than or equal to; Kg: Kilogram; **m^2^**: meter square

**Table 3:** Past medical history of study population

Variable	Frequency	Percentage
	N=200	100%
**Family history of diabetes mellitus**		
Yes	75	37.5%
No	113	56.5%
Don’t know	13	06.5%
**History of Chronic Illnesses**		
HIV positive	13	06.5%
Hypertension	03	01.5%
No	164	92.0%
**History of congenital malformations**		
Yes	02	01.0%
No	198	99.0%

**HIV**: human immunodeficiency virus

### Determinants of gestational diabetes mellitus

#### Socio-demographic characteristics

The mean age of participants with GDM compared with those without was 30.4 (SD 5.5) years versus 27.1 (SD 5.6) years (P=0.0009). In univariate analysis, GDM was associated with advanced maternal age >30 years (p=0.008), high BMI (p<0.001), past history of unexplained stillbirth (p<0.001), history of macrosomia (p<0.001) and family history of diabetes mellitus (p=0.01) (Table 4). In bivariate analysis, factors associated with GDM were advanced maternal age ≥30 years (OR 3.4: 95%CI 1.7-7.7, p=0.0004); high BMI ≥30 kg/m^2^(OR 6.2: 95% CI 2.9-13.1, p<0.001); history of unexplained stillbirth (OR 5.7: 95%CI 2.5-12.9, p<0.001) and history of macrosomia ≥4000g (OR 8.5: 95% CI 3.8-19, p<0.001) (Table 5).

**Table 4:** Unilabiate analysis of socio-demographics determinants of gestational diabetes mellitus among respondents

Variable	Gestational Diabetes Mellitus N (%)	No Gestational Diabetes Mellitus N (%)	Total N=200	P-value
**Maternal age (years)**				
16-20	0	18 (100%)	18 (100%)	**0.008**
21-25	7 (11.1%)	56 (88.9%)	63 (100%)	
26-30	16 (25.8%)	46 (74.2%)	62 (100%)	
31-45	15 (31.6%)	42 (68.4%)	57 (100%)	
**Occupation**				
Civil Servant	17 (85%)	3 (15%)	20 (100%)	>0.05
Housewife	35 (72.9%)	13 (27.1%)	48 (100%)	
Self employed	75 (78.9%)	20 (21.1%)	95 (100%)	
Unemployed	32 (86.5%)	5 (13.5%)	37 (100%)	
**Body Mass Index (Kg/m^2^)**				
18.5-24.9	23 (88.46%)	3 (11.54%)	26 (100%)	**<0.001**
25-29.9	95 (90.48%)	10 (9.52%)	105 (100%)	
≥30	41 (59.42%)	28 (40.58%)	69 (100%)	
**History of Unexplained stillbirth**				
Yes	16 (50%)	16 (50%)	32 (100%)	**<0.001**
No	25 (14.9)	143 (85.1%)	168 (100%)	
**History of macrosomia**				
Yes	16 (44.44%)	20 (55.56%)	36 (100%)	**<0.001**
No	143 (87.2%)	21 (12.8%)	164 (100%)	

**Kg**: Kilogram, **m^2^**: meter square

**Table 5:** Bivariate analysis of risk factors associated with gestational diabetes mellitus

Variable	Gestational Diabetes Mellitus N (%)	No Gestational diabetes Mellitus N (%)	OR (95% CI)	P-value
**Age ≥30 years**				
Yes	23 (34.8%)	43 (65.2%)	3.4 (1.7-7.0)	**<0.001**
No	18 (13.4%)	116 (86.6%)		
BMI ≥30 (kg/m^2^)				
Yes	8 (40.6%)	41 (59.4%)	6.2 (2.9-13.1)	**<0.001**
No	13 (9.9%)	118 (90.1%)		
**History of unexplained stillbirth**				
Yes	16 (50%)	16 (50%)	5.7 (2.5-12.9)	**<0.001**
No	25 (14.9%)	143 (85.1%)		
**History of macrosomia**				
Yes	20 (55.6%)	16 (44.4%)	8.5 (3.8-19)	**<0.001**
No	21 (12.8%)	14 (89.9%)		
**Family history of diabetes mellitus**				
Yes	25 (33.3%)	50 (66.7%)	5.5 (0.7-45)	0.08
No	1 (8.3%)	11 (91.7%)		

**OR:** odds ratio; **CI:** confidence interval; **BMI:** body mass index

**Kg:** kilogram; **m^2^:** meter square; **Y:** years

## Discussion

This study was carried out in three healthcare facilities at the Limbe Health District in Cameroon. The aims of the study were to determine the prevalence of GDM, establish the association between GDM and some socio-demographic variables, and compare the risk factors of GDM among pregnant women with GDM and those without GDM attending the selected healthcare facilities in the LHD. The mean age of study participants was 27.8 (SD 5.7) years and the prevalence of GDM was 20.5%. Majority, 13.5% participants had abnormal FPG alone while 3.5% participants had any two abnormal (PG) values. Most participants had secondary level education. Factors associated with GDM were advanced maternal age ≥30 years, high BMI ≥30 kg/m^2^, past history of unexplained stillbirth and history of macrosomia.

### Diagnosis of gestational diabetes mellitus

In this study we preferred to use the universal screening method because the selective screening method based on risk factors has scored poorly in detecting GDM [18]. Universal screening for GDM detects more cases and improves maternal and offspring prognosis compared to selective screening. The universal screening appears to be the most reliable and desired method for the detection of GDM, particularly in those populations with high risk for GDM. For universal screening, the test should be simple and cost effective [18, 19]. Furthermore, the two-step procedure of screening with 50g Glucose challenge test (GCT) and then diagnosing GDM based on Oral Glucose Tolerance Test (OGTT) is not feasible in a country like Cameroon because the pregnant women may have to visit the antenatal care clinic twice and at least three to five blood samples have to be drawn. This fact alone may reduce patients' compliance to follow-up [20-22]. The IADPSG diagnostic criterion is a one-step procedure, which has been able to clarify associations of maternal glucose lower than those diagnostic of diabetes mellitus. Moreover, it has increased the prevalence of GDM and optimized the predictability of adverse pregnancy outcomes in most studies [23-25]. Among the study participants, 13.5% had abnormal FPG alone while 3.5% participants had any two abnormal (PG) values. This was consistent with IADPSG diagnostic criteria which stipulate that there must be ≥1 abnormal PG level to be diagnostic of GDM [10]. However, since we did not do early pregnancy screening for DM, we could not ascertain for sure whether there was no undiagnosed preexisting diabetes mellitus (PEDM). This problem could have been solved by the HbA1c test which was out of the scope of this study because of elevated cost and lack of the DCCT/UKPDS standardized assay in the Limbe Health District [26].

### Prevalence of gestational diabetes mellitus

The prevalence of GDM in this study was 20.5%. GDM prevalence has been reported variably from 1.4 to 14% worldwide and differently among racial and ethnic groups. The prevalence is higher among Blacks, Latinos, Native Americans, and Asian women than in Caucasians [27]. Sobngwi *et al*. reported a prevalence of 5% to 17% in a study carried out in six regions of Cameroon [12]. One study reported a prevalence of GDM in some African countries to range from 0% in Tanzania to 13.9% in Nigeria. However, most of these studies used the WHO diagnostic criteria [28]. Another study in the United Arab Emirates by Agarwal *et al*. used the ADA, WHO and ADPIS (Australasian Diabetes in Pregnancy Society) criteria and had a prevalence of 14%, 20.6% and 23.1%, respectively [29]. The real prevalence of GDM is not known but varies from one geographic region to another. Secondly, the diagnostic criteria used by most studies are not similar [21, 30]. However, the high prevalence of GDM in our study may be attributed to the fact that the population under study falls within the high-risk group for diabetes mellitus (African). There may also have been some participants with undiagnosed preexisting diabetes mellitus (PEDM) as already highlighted. The IADPSG diagnostic criteria has also been reported to increase the prevalence of GDM in most studies because of the low threshold of diagnosis as compared to other diagnostic criteria [27, 31]. It also increased the Chinese population of women with GDM by 200% [32]. The current recommended time for the screening of GDM is between 24-28 weeks' gestation. Results of a FPG level must be available, followed by administration of a 75 g solution of anhydrous glucose load OGTT after overnight fasting. The IADPSG or WHO diagnostic criteria are mostly recommended. In this study, we used the IADPSG diagnostic criteria. Another study of 84 pregnant Cameroonian women using the IADPSG criteria reported that the 75 g OGTT is reproducible only in 74.2% pregnant women [14].

### Risk factors for gestational diabetes mellitus

Established risk factors for GDM in our study are advanced maternal age, obesity, past history of unexplained stillbirth and macrosomia. These are consistent with other studies [33, 34]. The increase in the prevalence of GDM in our study may be attributed to increased BMI, as high maternal weight is associated with a substantially higher risk of GDM [35]. It is noteworthy that the mean BMI in our study was 38.8 kg/m^2^, with 52% participants being overweight (BMI 25-29.9 Kg/m^2^) and 34.5% participants overtly obese (BMI>30 Kg/m^2^), which confirms that increased BMI is a risk factor for GDM [14]. However, there were very few women who lived a sedentary lifestyle to account for the high BMI and consequently GDM. It should be noted that Cameroonians over the years have become increasingly obese. This may be attributed to their dietary habits. Majority, eat and take alcoholic beverages in great quantities. The calorific value of their intake has hardly been quantified and the social valorization of stoutness exposes Cameroonians to obesity [36, 37]. This fact is contrary to the findings of Oken E *et al.*, who reported that physical activity, especially vigorous activity before pregnancy and at least light-to-moderate activity during pregnancy, may reduce risk for abnormal glucose tolerance and GDM [38]. Consistent with other studies, our study population had increased maternal age; 31% were in the age bracket 26-29 years while 28.5% were aged over 30. We did not have women above 36 years of age in our cohort. High parity was also a finding in this study [39]. Another study by Jang *et al*. reported that women with GDM were older, had higher pre-pregnancy weight, higher BMI, higher parities and higher frequencies of siblings with diabetes mellitus [40]. Amongst the risk factors identified in this study, overweight or obesity is the only modifiable risk factor. Moreover, there were 6.5% (13/200) HIV positive respondent in this study but we did not find any association between HIV and GDM. This is consistent with the study of Jao *et al*. 2013 at the Baptist Hospital Mutengene, Cameroon [13].

### Study limitations

The time frame used to undertake this study was short: 3 months. The sample size was too small to draw epidemiologic conclusions. Furthermore, we could not study other risk factors like PCOS because of additional cost. We were not able to obtain maternal weight gain in pregnancy because of the cross-sectional nature of the study. This study was done in three health facilities in the LHD. Conclusions from the study may not represent the actual prevalence of GDM in the whole Health District.

## Conclusion

The prevalence of GDM is high (20.5%). The risk factors associated with GDM identified were principally advanced maternal age (≥30 years), obesity, past history of unexplained stillbirths and history of macrosomia.

### What is known about this topic

- The criteria for the diagnoses of GDM are several and there is sometimes lack of concordance with results obtained by the different methods;
- The diagnosis of GDM in Cameroon is difficult because patients come late to/and are not very compliant with antenatal care visits;
- The Few studies in Cameroon reported a lower prevalence of GDM (5% to 17%) but no study had been carried out in the Limbe Health District.

### What this study adds

- The prevalence of GDM in this study is high (20%), and this may be associated with the IADPSG which has been able to clarify associations of maternal glucose lower than that diagnostic of diabetes mellitus;
- Obesity is the only modifiable risk factor of GDM identified among respondents in the Limbe Health District;
- There is need to organize early universal screening method for GDM amongst patients in the LHD to avoid adverse maternal and fetal outcomes especially as majority women at the LHD start antenatal care late and are sometimes irregular in their visits.

## Competing interests

The authors declare no competing interests.
